# Parent–offspring brain similarity: Specificities and commonalities among sex combinations–the TRIO study

**DOI:** 10.1016/j.isci.2025.112936

**Published:** 2025-06-19

**Authors:** Izumi Matsudaira, Ryo Yamaguchi, Yasuyuki Taki

**Affiliations:** 1 Frontier Research Institute for Interdisciplinary Sciences, Tohoku University, Sendai, Japan; 2 Smart-Aging Research Center, Tohoku University, Sendai, Japan; 3 Graduate School of Medicine, Tohoku University, Sendai, Japan; 4 Japan Society for the Promotion of Science, Tokyo, Japan

**Keywords:** Neuroscience, Cognitive neuroscience

## Abstract

Research suggests that parent–offspring brain similarities may underlie the intergenerational transmission of behavioral traits. However, most studies have focused on mothers and offspring, with few including fathers. This study aimed to extend our understanding of parent–offspring neural similarities by examining parent–offspring trios. The study included 152 Japanese biological trios consisting of adolescents or young adults and their fathers and mothers from the Transmit Radiant Individuality to Offspring (TRIO) study. We analyzed brain structural similarities across different parent–offspring sex combinations. Our findings confirmed that correlations in brain structural features were significantly stronger in parent–offspring dyads than between unrelated individuals. Notably, sons and daughters exhibited brain regions similar to their fathers only, mothers only, both, or neither. These results provide insights into genetic and environmental factors influencing brain development and aging across generations and could contribute to research on mechanisms underlying the intergenerational transmission of various traits.

## Highlights

• Neural similarity was detected in father–offspring and mother–offspring dyads
• Neural similarity varies depending on parent–offspring sex combinations
• Neural similarity partially relates to general intelligence similarity

## Graphical abstract

**Figure:**
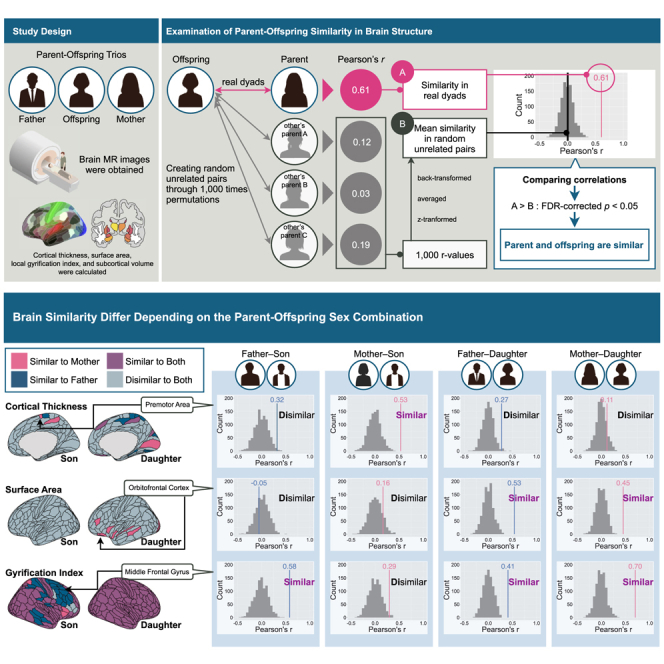

## Introduction

Intergenerational transmission describes the process by which parental traits influence offspring’s traits through genetic and/or nongenetic pathways.^1^ The association between parental mental illness and psychiatric symptoms in offspring is known as the intergenerational transmission of psychopathology.^2^^,^^3^^,^^4^ Intergenerational neuroimaging has been expected to elucidate the mechanism of this phenomenon.^5^ Several groundbreaking studies have used magnetic resonance imaging (MRI) to investigate the structural and functional brain similarities among parent–offspring pairs.^6^^,^^7^^,^^8^^,^^9^^,^^10^^,^^11^^,^^12^^,^^13^^,^^14^^,^^15^^,^^16^^,^^17^^,^^18^^,^^19^ Brain images are one of the endophenotypes of psychiatric disorders.^20^^,^^21^ Neuroimaging allows for the assessment of complex phenotypes, such as depression, bipolar disorder, and schizophrenia, using objective and quantifiable measures such as brain activity and morphology. However, to elucidate the mechanism of the intergenerational transmission of psychopathology, it is crucial to deepen our understanding of parent–offspring similarities in brain features while overcoming several challenges.

First, the differences in brain similarities between father–offspring and mother–offspring dyads remain unclear. Parent–offspring similarities have been reported in various brain features, such as in gray matter volume (GMV),^6^^,^^14^^,^^17^^,^^19^ cortical thickness (CT),^7^ surface area (SA),^16^^,^^17^ local gyrification index (LGI),^16^ sulcal morphology,^13^^,^^16^ white matter microstructure,^9^^,^^10^^,^^12^ neural metabolism,^11^ resting-state functional connectivity,^14^^,^^15^^,^^18^ and neural activation during reward processing.^8^ However, these studies have largely focused on mother–offspring dyads,^7^^,^^8^^,^^11^^,^^13^^,^^15^^,^^16^^,^^19^ whereas data on fathers, even when available, are typically limited.^9^^,^^10^^,^^12^^,^^14^^,^^18^ Genetic studies reported that genomic imprinting, an epigenetic phenomenon in which certain genes are expressed from either the maternally or paternally inherited allele,^22^ occurs more frequently in the brain than in somatic tissues.^23^^,^^24^ These parent-of-origin effects on brain development vary by offspring sex.^25^ Hence, it is essential to investigate neural similarities in both mother–offspring and father–offspring dyads. Although pioneering studies have investigated neural similarities across mother–daughter, mother–son, father–daughter, and father–son dyads,^6^^,^^17^ these studies lacked comparisons with unrelated adult–child pairs, leaving it unexamined whether the observed similarities are specific to parent-offspring dyads.^26^

Second, previous research generally investigated offspring in early childhood to adolescence,^9^^,^^12^^,^^14^^,^^15^^,^^16^^,^^18^^,^^19^ failing to clarify parent–offspring neural similarity at ages at which brain maturation has progressed. Brain maturation continues until adolescence, and structural indices undergo dynamic changes during this process.^27^^,^^28^^,^^29^^,^^30^ Additionally, most studies did not examine differences in parent–offspring similarity across various brain measures, despite their distinct developmental trajectories. Research on human brain development has primarily focused on CT, SA, subcortical volume (SV), and indices of cortical folding (gyrification). Each of these characteristics matures at different times,^31^^,^^32^^,^^33^^,^^34^ and has a different neuroanatomical mechanism^35^^,^^36^^,^^37^^,^^38^ and different genetic background.^39^^,^^40^^,^^41^ By simultaneously examining parent–offspring similarity in CT, SA, LGI, and SV within a single dataset, new insights can be gained into which specific features of each brain region develop under the influence of either the father or mother.

Third, the relationship between parent–offspring similarity in brain features and the similarity in behavioral indices has been little examined. To date, only one study has explored the association between mother–offspring neural and behavioral similarities, revealing that similarities in well-being are predicted by similarities in GMV in the anterior cingulate cortex (ACC) and prefrontal cortex.^19^ Previous studies on the intergenerational transmission of psychiatric disorders described the presence of homotypic continuity (parent and offspring have the same symptoms or diagnosis) and heterotypic continuity (parent and offspring display different symptoms or diagnoses).^1^ It remains unclear whether parent–offspring brain similarity reflects homotypic or heterotypic continuity. As psychiatric symptoms themselves are heterogeneosus,^42^ it would be valuable to examine parent–offspring similarity in cognitive function, which is considered a behavioral endophenotype,^20^^,^^43^ and investigate the relationship with similarity in brain features.

Considering the above, this study aimed to elucidate the specificities and commonalities in parent–offspring similarities of brain structural characteristics (CT, SA, LGI, and SV) among different parent–offspring sex combinations, using parent–offspring trios comprising offspring from adolescence to young adulthood and their biological parents.^44^ Additionally, we examined sex-specific differences in the behavioral similarities between parents and offspring, focusing on intelligence and personality traits that can be assessed using the same scale across a broad age range, to investigate whether neural similarity is reflected in the similarity of behavioral endophenotypes. Moreover, we tested the association between neural and behavioral similarities. We hypothesized that mother-specific or father-specific neural and behavioral similarities are observed in both sons and daughters.

## Results

### Age distributions of participants

**Figure 1:**
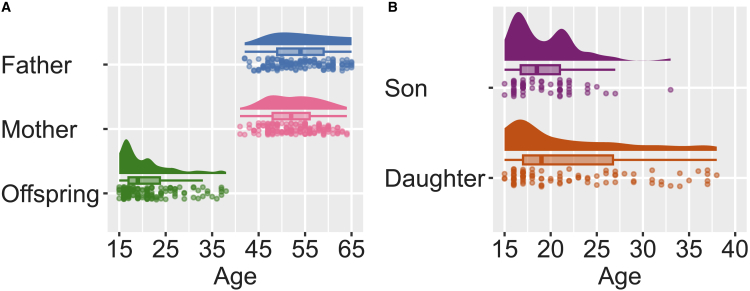
Age distributions of participants (A) Combined age distributions of fathers, mothers, and offspring. (B) Offspring age distributions separated by sex.

Descriptive statistics for participants’ age, intelligence, and personality traits are presented in Table 1. The age distributions of fathers, mothers, sons, and daughters are presented in Figure 1. Furthermore, the results of comparisons of the mean ages between various groups, including sons and daughters, the fathers and mothers of sons, the fathers and mothers of daughters, fathers with sons and fathers with daughters, and mothers with sons and mothers with daughters, are presented in Table 2.

**Table 1:** Characteristics of the study population

Father	Age	FSIQ	VCI	PRI	WMI	PSI	N	E	O	A	C
Mean	53.81	110.14	108.83	110.42	106.79	105.36	20.97	22.69	26.94	31.14	28.74
SD	6.09	13.04	12.55	13.75	15.19	14.17	8.62	6.00	4.92	5.20	5.25
Min	41	79	81	73	76	71	3	10	12	17	16
Max	65	135	132	137	144	143	46	35	41	47	42

Mother	Age	FSIQ	VCI	PRI	WMI	PSI	N	E	O	A	C
Mean	52.57	103.58	103.39	100.71	100.00	108.06	24.19	25.32	28.93	33.52	28.04
SD	5.48	10.64	10.76	12.00	11.63	13.77	8.13	6.38	5.29	4.79	6.61
Min	41	73	74	71	74	75	5	7	17	18	10
Max	64	133	132	130	141	145	46	41	43	46	44

Offspring	Age	FSIQ	VCI	PRI	WMI	PSI	N	E	O	A	C
Mean	21.07	106.74	108.80	103.05	105.99	102.52	28.75	25.28	27.90	30.23	25.59
SD	5.89	13.25	13.43	15.44	14.74	13.94	8.72	7.46	6.59	6.42	7.20
Min	15	71	74	69	71	73	5	8	14	12	7
Max	38	139	144	142	147	143	47	44	44	44	43

SD, standard deviation; FSIQ, full-scale intelligence quotient; VCI, verbal comprehension index; PRI, perceptual reasoning index; WMI, working memory index; PSI, processing speed index; N, neuroticism; E, extraversion; O, openness to experience; A, agreeableness; C, conscientiousness.

**Table 2:** Comparison of age among the study participants in relation to sons and daughters

	Age (mean)	t-value	df	*p*-value
Son	19.27	−46.61	147.58	<0.001
Daughter	22.17			
Son’s father	52.57	1.59	116.00	0.11
Son’s mother	50.98			
Daughter’s father	54.70	1.36	175.00	0.18
Daughter’s mother	53.51			
Son’s father	52.57	−2.11	143.00	0.04
Daughter’s father	54.70			
Son’s mother	50.98	−2.84	148.00	0.01
Daughter’s mother	53.51			

Student’s t test was performed to compare mean age between sons and daughters, son’s fathers and mothers, daughter’s fathers and mothers, son’s fathers and daughter’s fathers, and son’s mothers and daughter’s mothers. The homogeneity of variance across all pairs for each comparison was confirmed using Levene’s test. df, degrees of freedom.

### Neural similarities in father–offspring and mother–offspring dyads

**Figure 2:**
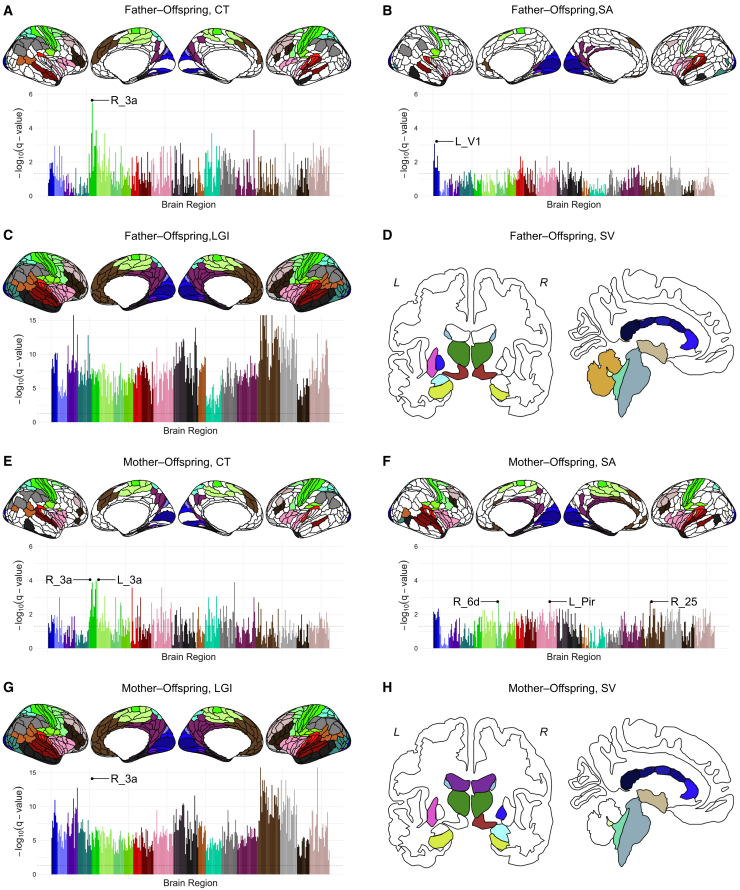
Neural similarities in father–offspring and mother–offspring dyads (A–H) Brain regions showing significantly stronger correlations in CT, SA, LGI, or SV in father–offspring or mother–offspring dyads compared to unrelated pairs. The original HCP-MMP1 atlas^45^ comprises 180 regions per hemisphere, which were grouped into 22 larger sections. The regions for which significantly stronger correlations were observed in father–offspring or mother–offspring dyads than in unrelated pairs are color-coded. The FDR-corrected *p*-values (*q*-values) for the 360 brain regions are presented in the plot. The vertical axis of the plot represents the log-transformed *q*-values. The horizontal dashed line represents the significance threshold of a *q*-value < 0.05. Each bar represents an individual brain region, and the colors of the bars correspond to those in the brain illustration. Subcortical regions in which significantly stronger correlations were observed between parents and offspring than between unrelated pairs are color-coded according to the ggseg plotting tool.^46^ CT, cortical thickness; LGI, local gyrification index; SA, surface area; SV, subcortical volume, L, left hemisphere; R, right hemisphere. See also Figures S1 and S2.

Neural similarities in father–offspring and/or mother–offspring dyads were observed across various brain regions. Figure 2 illustrates the brain regions in which neural similarity exceeded chance levels (FDR-corrected *p*-value [*q* value] < 0.05), indicating a significant difference in correlation coefficients between parent–offspring dyads and unrelated pairs. Detailed results for all brain regions are provided in Tables S1, S2, S3, S4, S5, S6, S7, and S8.

Notably, CT analysis revealed significantly greater similarity compared with unrelated pairs in 151 of 360 regions in father–offspring pairs and in 146 of 360 regions in mother–offspring pairs (Tables S1 and S5). A strong similarity was observed in bilateral area 3a (primary somatosensory cortex) for father–offspring (left; Pearson’s *r* in real dyads = 0.523, averaged Pearson’s *r* in unrelated pairs = 0.0002, *z* = 4.884, *q* < 0.001, right; Pearson’s *r* in real dyads = 0.593, averaged Pearson’s *r* in unrelated pairs = −0.0007, *z* = 5.753, *q* < 0.001) and mother–offspring dyads (left; Pearson’s *r* in real dyads = 0.543, averaged Pearson’s *r* in unrelated pairs = −0.003, *z* = 5.253, *q* < 0.001, right; Pearson’s *r* in real dyads = 0.523, averaged Pearson’s *r* in unrelated pairs = −0.0009, *z* = 4.990, *q* < 0.001).

For SA, 67 of 360 regions in father–offspring pairs and 157 of 360 regions in mother–offspring pairs displayed significantly greater similarity than observed in unrelated pairs (Tables S2 and S6). Notably, the bilateral visual cortex, insular cortex, and frontal operculum exhibited similar neural features in both father–offspring and mother–offspring dyads. Additionally, mother–offspring dyads featured particularly strong similarities in the somatosensory cortex and cingulate cortex.

LGI analyses revealed major similarities in father–offspring and mother–offspring dyads in almost all regions (Tables S3 and S7). The sole exception was the left 7AL (lateral anterior part of the somatosensory association cortex) in father–offspring dyads (Pearson’s *r* in real dyads = 0.219, averaged Pearson’s *r* in unrelated pairs = −0.0006, *z* = 1.885, *q* = 0.059), which did not exhibit a significant difference in correlation coefficients versus unrelated pairs.

In SV analyses, the similarity in the volumes of the bilateral caudate, thalamus, hippocampus, right ventral DC, left putamen, corpus callosum, third and fourth ventricles, brain stem, cerebellum cortex, and cerebellum white matter were common between father–offspring and mother–offspring dyads (Tables S4 and S8).

### Differences in neural similarities based on parent–offspring sex combinations

**Figure 3:**
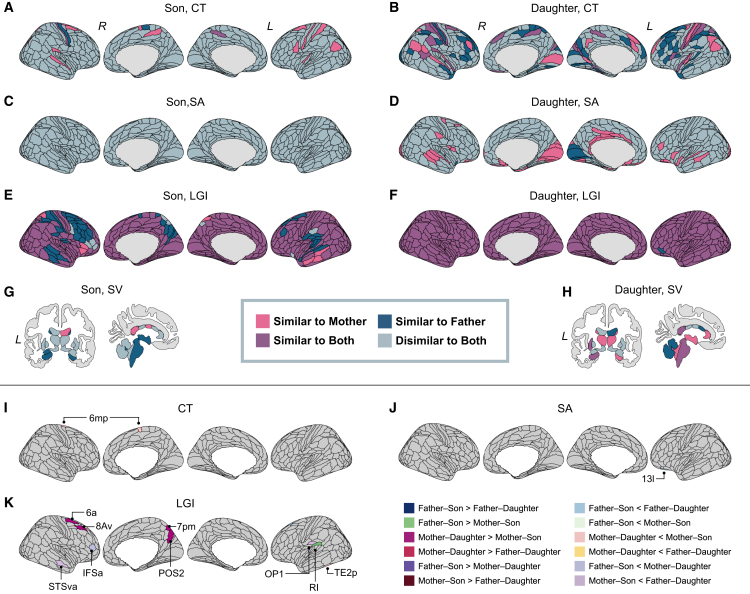
Differences in neural similarities based on parent–offspring sex combinations (A–H) Brain regions show parent–offspring neural similarity, separately for sons and daughters, are color-coded based on the comparison of correlation coefficients with unrelated pairs: pink, significant only in mother–offspring pairs; navy blue, significant only in father–offspring pairs; purple, significant in both mother– and father–offspring pairs; gray, nonsignificant in both. (I–K) Brain regions show significant differences in parent–offspring similarity across sex combinations, based on pairwise comparisons of correlation coefficients. The colors were assigned according to the contrast between sex combinations. CT, cortical thickness; SA, surface area; LGI, local gyrification index; SV, subcortical volume, L, left hemisphere; R, right hemisphere. See also Tables S9, S10, S11, S12, S13, S14, S15, S16, S17, S18, S19, S20, S21, S22, S23, and S24.

Based on the analysis of father–son, father–daughter, mother–son, and mother–daughter dyads (Figures S1 and S2; Tables S9, S10, S11, S12, S13, S14, S15, S16, S17, S18, S19, S20, S21, S22, S23, and S24), regions were colored to indicate those in which sons and daughters displayed similarity exclusively with the mother, exclusively with the father, with both parents, or with neither parent (Figures 3A–3H). Regarding CT, daughters exhibited a balanced distribution of regions resembling either one or both parents, whereas sons predominantly displayed similarity with their mothers (Tables S17 and S21). For SA, sons notably did not exhibit similarity with either parent in any region (Tables S10 and S14). For LGI, numerous brain regions exhibited similarity with both parents, regardless of the offspring’s sex. Daughters displayed similarity to both parents across almost all regions (Tables S19 and S23), whereas in sons, similarity was observed only with the father in several regions (Tables S11 and S15).

Figures 3I–3K presents the result of the pairwise comparison of correlation coefficients among different sex combinations. The statistical data from this analysis are summarized in Table 3.

**Table 3:** Result of the pairwise comparisons of correlation coefficients among different sex combinations

Feature	Region	Dyad 1	Dyad 2	r_Dyad1	r_Dyad2	*p*-value
CT	Right 6mp	Father–son	Father–daughter	0.321	0.275	0.772
		Father–son	Mother–son	0.321	**0.532**	0.169
		Mother–daughter	Mother–son	0.116	**0.532**	**0.005**
		Mother–daughter	Father–daughter	0.116	0.275	0.278
		Father–son	Mother–daughter	0.321	0.116	0.209
		Mother–son	Father–daughter	**0.532**	0.275	0.071
SA	Left 13L	Father–son	Father–daughter	−0.056	**0.539**	**<0.001**
		Father–son	Mother–son	−0.056	0.164	0.240
		Mother–daughter	Mother–son	**0.452**	0.164	0.059
		Mother–daughter	Father–daughter	**0.452**	**0.539**	0.451
		Father–son	Mother–daughter	−0.056	**0.452**	**0.002**
		Mother–son	Father–daughter	0.164	**0.539**	0.011
LGI	Left s6-8	Father–son	Father–daughter	0.079	**0.606**	**<0.001**
		Father–son	Mother–son	0.079	0.252	0.345
		Mother–daughter	Mother–son	**0.472**	0.252	0.136
		Mother–daughter	Father–daughter	**0.472**	**0.606**	0.211
		Father–son	Mother–daughter	0.079	**0.472**	0.012
		Mother–son	Father–daughter	0.252	**0.606**	0.009
	Right 6a	Father–son	Father–daughter	**0.451**	**0.407**	0.755
		Father–son	Mother–son	**0.451**	0.090	0.036
		Mother–daughter	Mother–son	**0.531**	0.090	**0.003**
		Mother–daughter	Father–daughter	**0.531**	**0.407**	0.298
		Father–son	Mother–daughter	**0.451**	**0.531**	0.543
		Mother–son	Father–daughter	0.090	**0.407**	0.046
	Right 8Av	Father–son	Father–daughter	**0.581**	**0.407**	0.181
		Father–son	Mother–son	**0.581**	0.297	0.058
		Mother–daughter	Mother–son	**0.704**	0.297	**0.001**
		Mother–daughter	Father–daughter	**0.704**	**0.407**	**0.004**
		Father–son	Mother–daughter	**0.581**	**0.704**	0.221
		Mother–son	Father–daughter	0.297	**0.407**	0.462
	Right POS2	Father–son	Father–daughter	**0.538**	**0.454**	0.522
		Father–son	Mother–son	**0.538**	0.160	0.020
		Mother–daughter	Mother–son	**0.588**	0.160	**0.003**
		Mother–daughter	Father–daughter	**0.588**	**0.454**	0.228
		Father–son	Mother–daughter	**0.538**	**0.588**	0.671
		Mother–son	Father–daughter	0.160	**0.454**	0.055
	Right 7p.m.	Father–son	Father–daughter	0.345	**0.456**	0.448
		Father–son	Mother–son	0.345	0.084	0.145
		Mother–daughter	Mother–son	**0.578**	0.084	**0.001**
		Mother–daughter	Father–daughter	**0.578**	**0.456**	0.272
		Father–son	Mother–daughter	0.345	**0.578**	0.082
		Mother–son	Father–daughter	0.084	**0.456**	0.018
	Left RI	Father–son	Father–daughter	**0.632**	**0.605**	0.798
		Father–son	Mother–son	**0.632**	0.231	**0.007**
		Mother–daughter	Mother–son	**0.493**	0.231	0.074
		Mother–daughter	Father–daughter	**0.493**	**0.605**	0.292
		Father–son	Mother–daughter	**0.632**	**0.493**	0.233
		Mother–son	Father–daughter	0.231	**0.605**	**0.007**
	Left OP1	Father–son	Father–daughter	**0.483**	**0.544**	0.636
		Father–son	Mother–son	**0.483**	0.147	0.045
		Mother–daughter	Mother–son	**0.525**	0.147	0.011
		Mother–daughter	Father–daughter	**0.525**	**0.544**	0.862
		Father–son	Mother–daughter	**0.483**	**0.525**	0.747
		Mother–son	Father–daughter	0.147	**0.544**	**0.007**
	Right IFSa	Father–son	Father–daughter	0.207	**0.438**	0.133
		Father–son	Mother–son	0.207	0.241	0.849
		Mother–daughter	Mother–son	**0.596**	0.241	0.010
		Mother–daughter	Father–daughter	**0.596**	**0.438**	0.157
		Father–son	Mother–daughter	0.207	**0.596**	**0.006**
		Mother–son	Father–daughter	0.241	**0.438**	0.191
	Right STSva	Father–son	Father–daughter	**0.522**	**0.665**	0.201
		Father–son	Mother–son	**0.522**	0.334	0.219
		Mother–daughter	Mother–son	**0.351**	0.334	0.908
		Mother–daughter	Father–daughter	**0.351**	**0.665**	**0.005**
		Father–son	Mother–daughter	**0.522**	**0.351**	0.218
		Mother–son	Father–daughter	0.334	**0.665**	**0.008**
	Left TE2p	Father–son	Father–daughter	**0.751**	**0.732**	0.812
		Father–son	Mother–son	**0.751**	**0.753**	0.975
		Mother–daughter	Mother–son	**0.472**	**0.753**	**0.006**
		Mother–daughter	Father–daughter	**0.472**	**0.732**	**0.006**
		Father–son	Mother–daughter	**0.751**	**0.472**	**0.007**
		Mother–son	Father–daughter	**0.753**	**0.732**	0.784

CT, cortical thickness; SA, surface area; LGI, local gyrification index. r_Dyad1 and r_Dyad2 indicate the correlation coefficients of each real parent–offspring dyad. Bold values in r_Dyad1 and r_Dyad2 indicate that the correlation coefficient in real parent–offspring dyads was significantly greater than that in unrelated pairs; in other words, the similarity in the feature of the brain region was confirmed by the brain similarity analysis. *P*-values highlighted in bold indicate that they are below the statistical threshold (*p* < 0.0085). The brain region names followed the notation in Glasser et al.^45^

For CT, mother–son dyads exhibited significantly greater correlation coefficients in CT of the right 6mp (posterior medial part of the supplementary motor area [SMA]) than mother–daughter dyads.

For SA, father–daughter and mother–daughter dyads displayed significantly greater correlation coefficients in SA of the left 13l (lateral subdivision of the posteromedial part of the orbital gyri) than father–son dyads.

For LGI, several regions exhibited significant differences in correlations among the combinations. In the left s6-8 (superior transition part of Brodmann area 6–8), the correlation was significantly stronger in father–daughter dyads than in father–son dyads. In the left TE2p (inferior temporal sulcus posterior), the correlation was significantly weaker in mother–daughter dyads than in the other combinations. In the left OP1 (operculum parietale 1), the correlation was significantly weaker in mother–son dyads than in father–daughter dyads, and the same result was recorded in the left RI (retroinsular cortex) and right STSva (ventral anterior bank of the superior temporal sulcus). Additionally, mother–son dyads exhibited a significantly weaker correlation than mother–daughter dyads in the right 6a (premotor cortex), 7 pm (medial posterior part of the somatosensory association cortex), 8Av (ventral part of the middle frontal gyrus [MFG]), and POS2 (parieto-occipital sulcus area 2). Meanwhile, mother–son dyads featured a significantly weaker correlation than father–son dyads in the left RI. Lastly, father–son dyads had a significantly weaker correlation than mother–daughter dyads in the right IFSa (anterior part of the inferior frontal sulcus).

For SV, no regions exhibited a significant difference in volume correlation coefficients among different parent–offspring sex combinations.

### Association between neural similarities and behavioral similarities

**Figure 4:**
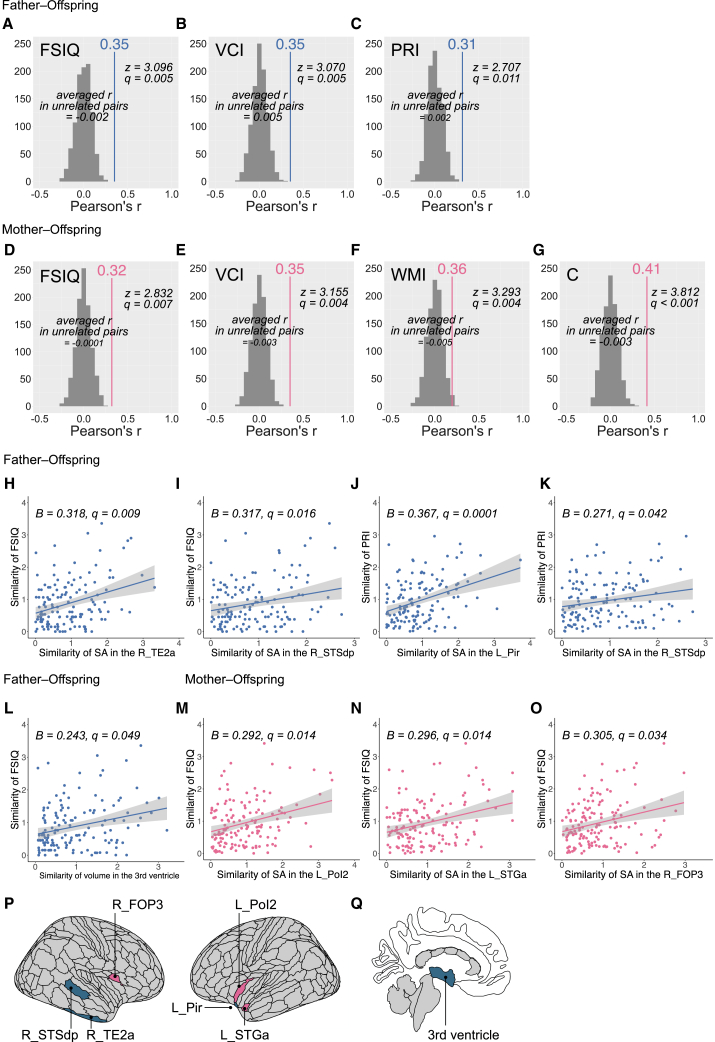
Association between neural similarities and behavioral similarities (A–G) The differences in correlation coefficients between real parent–offspring dyads and unrelated pairs. Gray histograms represent the distribution of correlation coefficients for 1,000 patterns of unrelated pairs. “Averaged *r* in unrelated pairs” denotes Z-transformed, averaged, and back-transformed correlation coefficients of unrelated pairs. The correlation coefficients for real parent–offspring dyads are presented as solid lines, with the corresponding values labeled above the lines. FSIQ, full-scale intelligence quotient; VCI, verbal comprehension index; PRI, perceptual reasoning index; WMI, working memory index; C, conscientiousness. See also Figures S3–S8; Tables S25, S26, S27, S28, S29, S30, S31, S32, S33, S34, S35, and S36. (H–O) Scatterplots illustrate the relationship between the Euclidean distance for FSIQ and that for each brain region in father–offspring and mother–offspring dyads. The standardized partial regression coefficients and FDR-corrected *p*-values (*q*-values) are displayed in the top left corner of each panel. SA, surface area; L, left hemisphere; R, right hemisphere. (P) Brain regions where the similarity in SA was significantly associated with FSIQ similarity in father–offspring (blue) and mother–offspring (pink) dyads. (Q) Brain region where the similarity in volume was significantly associated with FSIQ similarity in father–offspring dyads (blue).

Figures 4A–4G illustrates the intelligence and personality traits with significant parent–offspring correlations that exceeded those of unrelated pairs (Figures S3–S8; Tables S25, S26, S27, S28, S29, S28, S29, S30, S31, S32, S33, S34, S35, and S36). Similarities in FSIQ, VCI, and PRI were observed between fathers and their offspring (Figure S3). The significance of these similarities in FSIQ and VCI was maintained in father–son dyads but not in father–daughter dyads (Figures S4 and S5). Similarities in FSIQ, VCI, WMI, and conscientiousness were observed in mother–offspring dyads (Figure S6). In the sex-divided analysis, similarities in FSIQ, VCI, and WMI were only observed in mother–son dyads (Figure S7). Conversely, similarity in conscientiousness was only observed in mother–daughter dyads (Figure S8).

Figures 4H–4O illustrates the relationships between neural similarities and behavioral similarities. Notably, the similarity in FSIQ in father–offspring dyads was significantly and positively predicted by similarities in the volume of the third ventricle as well as the SA of the right TE2a (inferior temporal sulcus anterior) and STSdp (dorsal posterior bank of the superior temporal sulcus) (Figures 4P and 4Q). The similarity in PRI in father–offspring dyads was significantly predicted by similarities in the SA of the left Pir (piriform cortex) and the right STSdp (Figure 4P). The similarity in FSIQ in mother–offspring dyads was significantly predicted by similarities in the SA of the left PoI2 (posterior insular area 2), STGa (superior temporal gyrus anterior), and right FOP3 (frontal opercular area 3) (Figure 4P). However, when analyzing specific parent–offspring sex combinations, no significant associations were observed between neural and behavioral similarities.

## Discussion

We investigated the parent–offspring similarity in brain structure using a trio sample consisting of adolescence or young adults and their biological fathers and mothers. This study suggests that sex-specific patterns of parent–offspring similarity may exist in certain brain regions. In some regions, SA or LGI were similar between daughters and parents, but not between sons and parents. Additionally, there were regions where LGI resembled between daughters and parents or between sons and fathers, but not between sons and mothers. Conversely, regions where only mother–son dyads exhibited similarity in CT were also identified. Also, we examined the parent-offspring similarity in cognitive function. Our findings indicate that global intelligence is similar between both father–offspring and mother–offspring dyads, and that this similarity is associated with similarity in SA in several brain regions. To the best of our knowledge, this is the first study to demonstrate the variation of parent-offspring brain similarity among different sex combinations and to reveal the association between neural similarity and behavioral similarity.

Previous studies suggested the existence of matrilineal transmission patterns in specific brain structures, such as GMV in the ACC and orbitofrontal cortex (OFC) and SA in the caudal MFG.^6^^,^^17^ In our sample, SA in the left 13l, which is identical to the OFC,^47^ was similar between mothers and daughters and between fathers and daughters. Cortical GMV is a measure of the combination of CT and SA, and the statistical results of GMV are mainly influenced by SA.^48^ Therefore, the current results partially align with previous findings that the structure of the OFC is similar between mothers and daughters. However, because similarity was also observed between fathers and daughters in our sample, it is possible that previous findings were not specific to mother–daughter dyads. This unique finding was obtained by comparing parent–offspring sex combinations using trio samples. However, the possibility of daughter-specific similarity cannot be discounted. The pairwise comparison results revealed that for the SA of 13l, the correlation coefficient between fathers and daughters was significantly larger than that between fathers and sons.

Similar to the SA of 13l, several regions in which daughters, but not sons, resembled their fathers and mothers were identified. Specifically, the LGI of the left s6-8 exhibited a smaller correlation coefficient between fathers and sons than between fathers and daughters, whereas the LGI of the right 7 pm and left IFSa tended to have a smaller correlation coefficient between mothers and sons than between mothers and daughters. These differences in similarity could be attributable to the offspring’s sex. Previous studies investigating whether parenting styles differ according to the child’s sex reported inconsistent findings. It has been suggested that the difference in the parental use of controlling behavior and supportiveness for boys versus girls is minimal.^49^ However, a recent meta-analysis reported a tendency for parents to prefer daughters over sons, although this preference was not sufficiently obvious to be recognized by children.^50^ It has also been reported that fathers interact with their children in different manners, and their brain activity in response to their sons’ and daughters’ facial expressions has been found to differ.^51^ Therefore, regardless of the parents' or offspring’s awareness of it, there might be differences in how sons and daughters interact with their parents. It remains to be determined whether some shared factor in parent–offspring interactions causes the similarity in LGI between daughters and their parents or whether the similarity in LGI leads to differences in the relationships between daughters/sons and parents. Further investigation is needed.

The LGI in the right 6a, 8Av, POS2, STSva, left RI, and OP1 exhibited no similarity in mother–son dyads, and it was confirmed that the correlation coefficient for mother–son dyads was significantly smaller than that of at least one of the other combinations. Given that similarity was observed in father–son dyads, it is unlikely that the inability to detect a significant correlation coefficient in mother–son dyads was attributable to the small sample size of sons. Furthermore, because the correlation coefficients did not differ among father–son, father–daughter, and mother–daughter dyads, it can be interpreted that the lack of similarity is specific to mother–son dyads. These regions are heterogeneous both functionally and cytoarchitecturally, sharing no notable commonalities. However, as these regions include areas near primary sulci such as the parieto-occipital, central, and superior temporal sulci, it is possible that genetic influences are strongly reflected in the structure of these areas.

One possible explanation for the current results is the presence of a sex-specific parent-of-origin effect in genes involved in cortical gyrification, in which alleles inherited from the mother do not function in male offspring. In primary sulci, the boundaries of cytoarchitecture are believed to correspond to the characteristics of cortical folding.^52^^,^^53^^,^^54^ This idea is based on the hypothesis that a larger ratio of upper-layer neurons to lower-layer neurons makes cortical gyrification more likely to occur.^55^ Genes such as *FGF*,^55^ *Trnp1*,^56^ and *ARHGAP11B*^57^ are involved in the production of upper-layer neurons and the tangential expansion of the cortical surface. However, no reports have suggested that any of these genes are subject to imprinting. Therefore, the possibility that genes affecting cortical gyrification through their impact on cortical layer structure are regulated by expression control via imprinting is low, and this cannot explain our observation of no similarity in LGI in mother–son dyads.

Another possibility is the involvement of both pre- and postnatal environmental factors that influence cortical gyrification. Reports have described the associations of cortical gyrification with factors such as the intrauterine environment^58^ and familial socioeconomic status.^59^ As genetic factors may influence the intrauterine environment,^60^ the environment to which offspring is exposed in the mother’s womb might be partially similar to that to which the mother was exposed during her own fetal period. Experiencing a similar intrauterine environment could result in similarities in the developmental processes of cortical gyri, which develop early. Additionally, it has been suggested that the effects of the intrauterine environment on the brain can differ depending on the offspring’s sex.^61^ Therefore, the finding that the LGI of certain regions is similar in mother–daughter dyads but not in mother–son dyads might be related to sex differences in the effects of the prenatal environment on the brain. This also raises the possibility that the similarity in LGI observed in father–son and father–daughter dyads is realized through mechanisms entirely different from those underlying the similarity in mother–daughter dyads. An association between the father’s socioeconomic status and the fetal gyrification index has been reported.^59^ As the intergenerational transmission of socioeconomic status has been widely reported,^62^^,^^63^ fathers might have experienced similar economic conditions during their childhood as those experienced by their sons or daughters. Therefore, it is speculated that the similar experience of socioeconomic status during childhood contributes to the similarity in LGI between fathers and their offspring, regardless of the offspring’s sex.

In summary, the similarity in gyrification might be influenced by similar early life experiences between parents and their offspring. Furthermore, the common environmental factors influencing similarity between parents and their offspring could differ between father–offspring and mother–offspring pairs.

There were also regions in which only mother–son dyads exhibited strong feature correlations that were sufficiently distinct from those of unrelated individuals. The pairwise comparison revealed that the only region in which a significant difference in correlation coefficients was observed compared with other pairs was the CT of the left 6mp, which was not observed in father–son, father–daughter, and mother–daughter pairs. The 6mp, which is part of the SMA, is responsible for preparing complex motor actions.^45^^,^^64^ The SMA is believed to participate in sensorimotor abnormalities observed in autism spectrum disorder (ASD).^65^^,^^66^ ASD has a higher prevalence in males, and research is underway to explore the underlying genetic mechanisms.^67^^,^^68^ Recent studies have suggested the involvement of mitochondrial DNA (mtDNA), which is exclusively inherited from the mother, in the pathology of ASD.^69^^,^^70^ The brain requires a substantial amount of mitochondrial energy, which is utilized for processes such as synapse formation.^71^ Therefore, mtDNA is considered an important factor in the development of CT. Considering this background, mtDNA inherited from the mother by the son might be involved in the similarity of CT in the SMA in mother–son dyads. However, because our sample did not include individuals diagnosed with ASD, this interpretation remains speculative. In cohort studies that did not target specific disorders, the association between mtDNA and psychiatric traits was inconsistent.^72^^,^^73^ Further research is needed to clarify the relationship between mtDNA and brain development.

Given that general cognitive ability is known to be more strongly driven by SA than by CT,^74^ it is reasonable that the similarity in FSIQ was associated with that in SA in certain regions. In all regions in which the association between SA similarity and FSIQ or PRI similarity was confirmed, the involvement of cognitive functions contributing to FSIQ and PRI has been reported. Specifically, FOP3 has been reported to play a role in selective attention,^75^ STGa has been associated with auditory short-term memory,^76^ PoI2 has been linked to the monitoring of behavior,^77^ TE2a has been implicated in language comprehension,^78^ STSdp has been found to contribute to audiovisual information integration,^79^ and Pir has been shown to be involved in hippocampus-dependent associative memory retrieval.^80^ The similarity in SA among these regions might reflect the similarity in intelligence between parents and their offspring. Additionally, in father–offspring dyads, the similarity in the size of the third ventricle was also significantly associated with the similarity in FSIQ. Enlargement of the third ventricle has been frequently reported in various psychiatric and neurological disorders, such as schizophrenia and Parkinson’s disease.^81^^,^^82^ Therefore, the third ventricle could represent a biomarker of general cognitive ability, and similarity in its size could reflect similarity in intelligence. Consequently, our study demonstrated that similarity in brain structure is associated with similarity in cognitive functions, which serve as behavioral endophenotypes. However, when analyzing offspring by sex, this association was not observed, which might be attributable to the relatively small sample sizes (60 sons and 90 daughters) rather than indicating a genuinely sex-independent relationship. As the correlation coefficients for intelligence between parents and offspring were lower than 0.4, this relationship does not appear to represent a strong effect. For most brain regions, the relationship between similarity in features in parent–offspring dyads to behavioral endophenotypes remains unclear. In this study, similarities were treated equally regardless of whether the parent–offspring values were close on the higher or lower side of the average, resulting in a mixture of qualitatively different types of similarity. Further study is needed to determine whether it is appropriate to assume a linear relationship in which greater similarity in brain structure corresponds to greater similarity in other endophenotypes.

In conclusion, our study using Japanese trio samples confirmed the existence of similarities in brain structure and behavior exist between parents and offspring that exceeded those found between unrelated individuals. Furthermore, we discovered that whether specific brain regions and features resemble the father, the mother, both, or neither varies depending on the offspring’s sex. Our findings are expected to contribute to the investigation of the validity of using parent–offspring brain similarity as an endophenotype for the intergenerational transmission of psychiatric disorders.

### Limitations of the study

Some limitations of this study must be considered while interpreting the findings. First, the differing sample sizes between sons and daughters could have affected the detection of sex-specific effects. Overall, especially concerning CT and SA, more regions resembling parents were identified in daughters than in sons. However, because the study included fewer sons than daughters, the possibility that existing parent–son correlations were not detected as statistically significant because of the sample sizes cannot be dismissed. Second, the wide age range among parents and offspring could have introduced age-related brain changes that cannot be fully controlled in statistical analysis, even using age as a covariate. As most offspring in this study were teenagers, future research should recruit more participants in their 20s and 30s and conduct age group-specific analyses. Addressing the first and second limitations requires careful consideration in the recruitment of participants. We distributed advertisements to high schools and universities, which made it easier to recruit adolescents and their parents. We attempted to recruit offspring aged 20 years and older and their parents by placing advertisements in local magazines, but this approach was not sufficient. To increase the participation of offspring aged 20 years and older and their parents, it might be necessary to enhance accessibility to potential participants by using public records such as the Basic Resident Register. Additionally, establishing a system that allows data collection at multiple sites would be necessary to facilitate the participation of parent–offspring trios who live apart. Finally, this study’s cross-sectional design limited the understanding of developmental trajectories. Long-term longitudinal studies are required to clarify when and how parent–offspring similarities in brain structure and behavior emerge and evolve.

## Resource availability

### Lead contact

Further information and requests for resources and reagents should be directed to and will be fulfilled by the lead contact, Izumi Matsudaira (izumi.matsudaira.e4@tohoku.ac.jp).

### Materials availability

This study did not generate new unique reagents.

### Data and code availability

• **Data:** This article does not report a standardized dataset. Our data are not publicly available as this study is part of an ongoing project. However, data supporting the results of this study will be provided upon request to the corresponding author.
• **Code:** The script used in this study was provided by a third party who is not one of the authors, and therefore it has not been made publicly available. However, it can be made available upon request by contacting the corresponding author.
• **Additional information**: Any additional information required to reanalyze the data reported in this article is available from the lead contact upon request. All requests for collaboration can also be directed to the corresponding author.

## Acknowledgments

This research received funding from a Grant-in-Aid for Young Scientists (Grant No. 22K13809), Grant-in-Aid for Transformative Research Areas (A) (Grant Nos. 22H05209 and 24H00896), and Grant-in-Aid for Scientific Research (B) (Grant no. 23K27258), provided by the Ministry of Education, Culture, Sports, and Technology (MEXT) and the Japan Society for the Promotion of Science (JSPS) to IM. Additionally, it was supported by the Program for the Creation of Interdisciplinary Research from the Frontier Research Institute for Interdisciplinary Sciences, Tohoku University, and a research grant from the Intelligent Cosmos Academic Foundation awarded to IM. Further support was provided by JST SPRING (Grant No. JPMJSP2114) and a Grant-in-Aid for JSPS Fellows (Grant No. 23KJ0220) to RY. YT was supported by a Grant-in-Aid for Scientific Research (B) (Grant no. 19H04211) from MEXT. The curation and analysis of neuroimaging data were funded by JSPS KAKENHI Grant Number JP22H04926 and the Grant-in-Aid for Transformative Research Areas— Platforms for Advanced Technologies and Research Resources “Advanced Bioimaging Support.” We wish to express our deepest thanks to the participants for their willingness to participate in this study. Special thanks are directed to Yukiko Suginome, Saeko Hoshi, Chieko Miura, Maiko Chiba, Misaki Abe, Junko Kato, and Shuzo Yamamoto for their invaluable support. We are also grateful to Dr. Ryosuke Kimura, Dr. Tadashi Imanishi, Dr. Hiroaki Tomita, Ayaka Uchiyama, Kanna Oyama, Mihiro Koizumi, Takumi Uchiyama, Yuka Aoki, Fumiaki Nitta, Yuka Hatayama, Megumi Kato, Maiko Suenaga, Jun Nomura, Ryuhei Ohgi, Ruri Takahashi, Yuto Tanaka, Saki Uchida, Ruriko Igarashi, and all other staff for their contributions to data collection. Additionally, we extend our gratitude to Drs. Kiyotaka Nemoto and Takuya Hayashi for their support with the neuroimaging analysis, and to Dr. Plamina Dimanova for sharing the MATLAB script for the permutation analysis. We thank all institutions that cooperated with us in recruiting study participants. Lastly, we would like to thank Enago (https://www.enago.jp/) for English language editing.

## Author contributions

I.M. and R.Y. designed the research. I.M. and R.Y. collected the data. I.M. and R.Y. analyzed the data. I.M., R.Y., and Y.T. acquired funding. Y. T. supervised this study. and I.M. wrote and edited the article. All authors read and approved the final version of the article.

## Declaration of interests

We used BrainSuite, developed by CogSmart, Inc., as an incentive for the participants. Y.T., who is the chief scientific officer of CogSmart, Inc., obtained approval from the Conflict of Interest (COI) Management Committee of Tohoku University for his involvement in this study. This study was also supported by the joint research fund of CogSmart, Inc., but these funds have not been used to pay for the use of BrainSuite. The remaining authors declare that the research was conducted in the absence of any commercial or financial relationships that could be construed as a potential COI.

## Declaration of generative AI and AI-assisted technologies in the writing process

During the preparation of this work, we used ChatGPT to make MATLAB and R scripts for statistical analysis. After using this this tool, we reviewed and edited the content as needed, and we take full responsibility for the content of the publication.

## STAR★Methods

### Key resources table

**Table:** 

REAGENT or RESOURCE	SOURCE	IDENTIFIER
**Software and algorithms**		

FreeSurfer	http://surfer.nmr.mgh.harvard.edu/	RRID: SCR_001847
Connectome Workbench	http://humanconnectome.org/connectome/connectome-workbench.html	RRID: SCR_008750
MATLAB R2022a	https://www.mathworks.com	RRID: SCR_001622
R version 4.3.1	http://www.r-project.org/	RRID: SCR_001905

### Experimental model and study participant details

#### Overview of the dataset

This research is part of the Transmit Radiant Individuality to Offspring (TRIO) study, an ongoing project.^44^ The TRIO study was launched in 2020 to elucidate the mechanisms of the association between intergenerational transmission and human brain development by focusing on biologically related parent–offspring trios consisting of a father, mother, and offspring. As of February 2025, the TRIO study includes data from 289 parent–offspring trios consisting of children aged 10–38 years and their parents. This project adhered to the principles enshrined in the Declaration of Helsinki^83^ and received approval from the Institutional Review Board of Tohoku University (Approval No. 2022- 1–534). Written informed consent was obtained from all participants before the study. For minors (aged <18 years), parental consent was also required. The detailed method for participant recruitment was described in our previous paper.^44^

To control for genetic background, participation was limited to Japanese individuals with no relatives within the third degree of kinship from other ethnicities. Individuals with a history of cerebrovascular disease, brain tumor, intracranial disease, degenerative brain disease, epilepsy, severe heart disease, and brain injury with impaired consciousness were not eligible for inclusion. These conditions were verified at the time of participation, and if any trio member met the exclusion criteria, their participation was declined.

This study focuses on a subsample of 152 parent–offspring trios, consisting of offspring aged 15 years and older and their parents. Following rigorous screening, two parent–offspring trios were excluded due to the offspring’s neurological disorders and suboptimal brain imaging quality. Additionally, five fathers were excluded from the study for the following reasons: two exceeded the upper age limit for inclusion, one had a neurological disorder, one had a brain hematoma, and one declined to undergo MRI scanning. Finally, 145 father–offspring dyads (58 sons and 87 daughters) and 150 mother–offspring dyads (60 sons and 90 daughters) were included in this study.

### Method details

#### MRI parameters

Brain images were acquired using a 3-Tesla dStream Achieva scanner (Philips Medical Systems, Best, Netherlands) equipped with a 20-channel head-neck coil. For each participant, two types of images were acquired. Sagittal T1-weighted images (T1WIs) were acquired using a magnetization-prepared rapid gradient-echo sequence with parameters including 368× 368 matrix, 11 ms repetition time, 5.1 ms echo time, 256 × 256 mm field of view, 257 slices, and 0.7 mm slice thickness. Sagittal T2-weighted images (T2WIs) were acquired using a spin-echo sequence with parameters including 368 × 368 matrix, 2500 ms repetition time, 3200 ms echo time, 256 × 256 mm field of view, 250 slices, and 0.7 mm slice thickness. Image quality was visually inspected immediately after imaging, and if significant motion artifacts were detected, re-imaging was performed.

#### Preprocessing of brain images

All neuroimaging data was preprocessed using FreeSurfer v7.3.2 to estimate CT, SA, and LGI of cortical regions, and SV.^84^^,^^85^^,^^86^ To enhance segmentation quality, three steps preceded the standard recon-all pipeline. First, AC-PC alignment was conducted using a 6-degree-of-freedom rigid body transformation.^87^ Second, the nonuniform intensity normalization was conducted using N4ITK.^88^ Third, brain extraction was performed using HD-BET.^89^ These three processes were applied to both T1WIs and T2WIs. Additionally, the brain mask image generated by HD-BET for T1WIs was utilized by incorporating the “-xmask” option in the “recon-all” process. As HD-BET removed extracerebral tissue, the “-noskullstrip” option was used to prevent skull stripping within the recon-all pipeline. Quality assurance of the preprocessing results was conducted through visual inspection and manual editing of the reconstructed images. Specifically, we verified that the boundaries between white and gray matter, as well as the pial surface, were accurately delineated. Any images with segmentation errors or topological defects underwent manual corrections, followed by a re-run of the recon-all process. CT, SA, and SV were obtained from the standard recon-all output. To obtain LGI, we performed an additional recon-all run with the “-localGI” flag.^86^ This process was performed additionally after the completion of quality assurance.

CT, SA, and LGI for each brain region were calculated based on the Human Connectome Project Multi-Modal Parcellation version 1.0 (HCP-MMP1) atlas.^45^ However, since the HCP-derived atlas and the FreeSurfer data are on different surface spaces (fs_LR mesh and fsaverage mesh, respectively), we resampled the HCP-MMP1 label map file downloaded from the BALSA database (https://balsa.wustl.edu/file/L632n)^90^ into individual surface data, using Connectome Workbench version 1.5.0. The resampling procedure was based on the document in the HCP Public Pages (https://wiki.humanconnectome.org/docs/assets/Resampling-FreeSurfer-HCP_5_8.pdf). Additionally, SV was calculated based on the Automatic Subcortical Segmentation (aseg) atlas.^85^

#### Intelligence and personality traits

Wechsler Adult Intelligence Scale Fourth Edition (WAIS-IV)^91^ was used to measure the participants’ global intelligence. For offspring aged 15, the Wechsler Intelligence Scale for Children Fourth Edition (WISC-IV)^92^ was used. Ten core subtests were conducted to calculate the full-scale intelligence quotient (FSIQ) along with four factors (verbal comprehension index [VCI]; perceptual reasoning index [PRI]; working memory index [WMI]; and processing speed index [PSI]). These five scores were used for the subsequent analysis. The tests were conducted in a quiet, distraction-free room, one-on-one with trained staff familiar with the procedure and participants.

Personality traits were assessed using the NEO five-factor inventory (NEO-FFI),^93^ a 60-item questionnaire that evaluates five personality traits (neuroticism, extraversion, openness to experience, agreeableness, conscientiousness) using a five-point Likert scale. A previous study has demonstrated the reliability and validity of the Japanese version of the NEO-FFI.^94^

### Quantification and statistical analysis

#### Brain similarity analysis

The analysis of parent–offspring brain similarity was conducted following the procedures suggested in previous studies.^16^^,^^19^ Here, we explain the process using CT similarity between mother and offspring as an example. At first, the CT values of individual brain regions were extracted, and Pearson’s correlation coefficients were calculated for each region between biological mother and offspring. Next, using MATLAB (MathWorks Inc., Natick, MA, USA), the mothers were randomly permuted, and new unrelated pairs were created by pairing each offspring with a different participants’ mother. This procedure was repeated 1,000 times. As a result, 1,000 datasets consisting of pairs of offspring and a different participants’ mother were generated. By calculating Pearson’s correlation coefficients for CT in each brain region for unrelated pairs in each of 1,000 sets, a normal distribution of correlation coefficients for unrelated individuals was generated. Using FisherZ() and FisherZInv() function implemented in the “DescTools” package in R, all 1,000 correlation coefficients were Fisher’s Z-transformed, averaged, and then back-transformed into a correlation coefficient yielding a similarity score for unrelated pairs. Finally, this score was compared to the correlation coefficient of real mother–offspring dyads. If the correlation coefficient of real dyads was significantly higher, the CT of that region was deemed similar between mother and offspring. The “cocor” package in R was used to test the differences in correlation coefficients.^95^

The same series of steps described above was performed for CT, SA, LGI, and SV in father–offspring and mother–offspring dyads. In the analysis of CT or LGI, age of the parent and offspring was controlled when calculating correlation. For SA and SV analyses, both age and total intracranial volume (TIV) of the parent and offspring were controlled when calculating correlation. For CT, SA, and LGI, similarity was examined in 360 regions based on the HCP-MMP1 atlas. For SV, similarity was assessed in 32 regions, including the hippocampus, amygdala, thalamus, basal ganglia, cerebellum, brainstem, ventricles, and corpus callosum. For multiple comparison correction in the test of correlation coefficients differences, the False Discovery Rate (FDR) was adjusted using the Benjamini-Hochberg (BH) method.^96^ A statistical threshold of FDR-corrected *p* (*q* value) < 0.05 was established to determine significant brain similarity between parents and offspring.

#### Comparison of brain similarity across sex combinations

After conducting the aforementioned analyses separately for father–offspring and mother–offspring pairs, we repeated them for each parent–offspring sex combination (father–son, father–daughter, mother–son, mother–daughter). After that, pairwise correlation coefficients were compared to examine the significance of differences in brain structural similarity across biological parent–offspring sex combinations using the Comparing_Multiple_Independent_Correlation_Coefficients() function implemented in the “DBM.functions” package in R.^97^ CT, SA, and LGI were tested across 360 regions, whereas SV was tested across 32 regions. Theoretically, six types of comparisons were possible: father–son vs. father–daughter, mother–son vs. mother–daughter, father–son vs. mother–son, father–daughter vs. mother–daughter, father–son vs. mother–daughter, and mother–son vs. father–daughter. The Comparing_Multiple_Independent_Correlation Coefficients() function implements Šidák’s correction to control the family-wise error rate.^97^^,^^98^ The adjusted significance level was set to 1 − (1–0.05)^1/6^ = 0.0085. Among the regions in which a significant difference in correlation coefficients was observed between sex combinations, some did not exhibit a significant difference from unrelated pairs in the similarity analysis (i.e., regions in which specific parent–offspring correlations were not confirmed). These regions were excluded from the report.

#### Behavioral similarity analysis

The similarity between parents and offspring in intelligence and personality traits was examined using the same procedure employed for brain similarity. The age of both parent and offspring were controlled for only in the analysis of personality traits. Parent–offspring dyads with missing scores for either the parent or offspring were excluded. The final sample size for the analysis of intelligence was 140 father–offspring dyads (56 sons, 84 daughters) and 147 mother–offspring dyads (59 sons, 88 daughters). For personality traits, the sample sizes were 142 father–offspring dyads (56 sons, 86 daughters) and 148 mother–offspring dyads (58 sons, 90 daughters). To account for multiple comparisons, the FDR was adjusted using the BH method, with a statistical threshold of *q* < 0.05. Analyses were conducted separately for father–offspring dyads, mother–offspring dyads, and each parent–offspring sex combination, mirroring the approach used in the neuroimaging analysis.

#### The association between brain similarity and behavioral similarity

The Euclidean distance between parent and offspring was used to measure the extent of similarity in brain regions and behavioral indices (intelligence and personality traits) where similarity was observed in the previous analysis. In both cases, the Euclidean distance was calculated using standardized values of the raw measurements. Multiple regression analysis was conducted using the Euclidean distance of behavioral indicators as a dependent variable, the Euclidean distance of brain features as the predictor, and the age of both parent and offspring as covariates. When SA and SV were used as predictors, the TIV of both the parent and offspring was added to the model as a covariate. For each of CT, SA, LGI, and SV, the FDR was adjusted using the BH method, with a statistical threshold set at *q* < 0.05.

## Supplemental information

Document S1. Figures S1–S8

Table S1. Results of the comparison of the correlation coefficients of cortical thickness in each region between father–offspring dyads and unrelated pairs, related to Figure 2

Table S2. Results of the comparison of the correlation coefficients of surface area in each region between father–offspring dyads and unrelated pairs, related to Figure 2

Table S3. Results of the comparison of the correlation coefficients of the local gyrification index in each region between father–offspring dyads and unrelated pairs, related to Figure 2

Table S4. Results of the comparison of the correlation coefficients of volume in each subcortical region between father–offspring dyads and unrelated pairs, related to Figure 2

Table S5. Results of the comparison of the correlation coefficients of cortical thickness in each region between mother–offspring dyads and unrelated pairs, related to Figure 2

Table S6. Results of the comparison of the correlation coefficients of surface area in each region between mother–offspring dyads and unrelated pairs, related to Figure 2

Table S7. Results of the comparison of the correlation coefficients of the local gyrification index in each region between mother–offspring dyads and unrelated pairs, related to Figure 2

Table S8. Results of the comparison of the correlation coefficients of volume in each subcortical region between mother–offspring dyads and unrelated pairs, related to Figure 2

Table S9. Results of the comparison of the correlation coefficients of cortical thickness in each region between father–son dyads and unrelated pairs, related to Figures 3, S1, and S2

Table S10. Results of the comparison of the correlation coefficients of surface area in each region between father–son dyads and unrelated pairs, related to Figures 3, S1, and S2

Table S11. Results of the comparison of the correlation coefficients of the local gyrification index in each region between father–son dyads and unrelated pairs, related to Figures 3, S1, and S2

Table S12. Results of the comparison of the correlation coefficients of volume in each subcortical region between father–son dyads and unrelated pairs, related to Figures 3, S1, and S2

Table S13. Results of the comparison of the correlation coefficients of cortical thickness in each region between mother–son dyads and unrelated pairs, related to Figures 3, S1, and S2

Table S14. Results of the comparison of the correlation coefficients of surface area in each region between mother–son dyads and unrelated pairs, related to Figures 3, S1, and S2

Table S15. Results of the comparison of the correlation coefficients of the local gyrification index in each region between mother–son dyads and unrelated pairs, related to Figures 3, S1, and S2

Table S16. Results of the comparison of the correlation coefficients of volume in each subcortical region between mother–son dyads and unrelated pairs, related to Figures 3, S1, and S2

Table S17. Results of the comparison of the correlation coefficients of cortical thickness in each region between father–daughter dyads and unrelated pairs, related to Figures 3, S1, and S2

Table S18. Results of the comparison of the correlation coefficients of surface area in each region between father–daughter dyads and unrelated pairs, related to Figures 3, S1, and S2

Table S19. Results of the comparison of the correlation coefficients of the local gyrification index in each region between father–daughter dyads and unrelated pairs, related to Figures 3, S1, and S2

Table S20. Results of the comparison of the correlation coefficients of volume in each subcortical region between father–daughter dyads and unrelated pairs, related to Figures 3, S1, and S2

Table S21. Results of the comparison of the correlation coefficients of cortical thickness in each region between mother–daughter dyads and unrelated pairs, related to Figures 3, S1, and S2

Table S22. Results of the comparison of the correlation coefficients of surface area in each region between mother–daughter dyads and unrelated pairs, related to Figures 3, S1, and S2

Table S23. Results of the comparison of the correlation coefficients of the local gyrification index in each region between mother–daughter dyads and unrelated pairs, related to Figures 3, S1, and S2

Table S24. Results of the comparison of the correlation coefficients of volume in each subcortical region between mother–daughter dyads and unrelated pairs, related to Figures 3, S1, and S2

Table S25. Results of the comparison of the correlation coefficients of intelligence between father–offspring dyads and unrelated pairs, related to Figures 4 and S3

Table S26. Results of the comparison of the correlation coefficients of intelligence between father–son dyads and unrelated pairs, related to Figures 4 and S4

Table S27. Results of the comparison of the correlation coefficients of intelligence between father–daughter dyads and unrelated pairs, related to Figures 4 and S5

Table S28. Results of the comparison of the correlation coefficients of intelligence between mother–offspring dyads and unrelated pairs, related to Figures 4 and S6

Table S29. Results of the comparison of the correlation coefficients of intelligence between mother–son dyads and unrelated pairs, related to Figures 4 and S7

Table S30. Results of the comparison of the correlation coefficients of intelligence between mother–daughter dyads and unrelated pairs, related to Figures 4 and S8

Table S31. Results of the comparison of the correlation coefficients of personality traits between father–offspring dyads and unrelated pairs, related to Figures 4 and S3

Table S32. Results of the comparison of the correlation coefficients of personality traits between father–son dyads and unrelated pairs, related to Figures 4 and S4

Table S33. Results of the comparison of the correlation coefficients of personality traits between father–daughter dyads and unrelated pairs, related to Figures 4 and S5

Table S34. Results of the comparison of the correlation coefficients of personality traits between mother–offspring dyads and unrelated pairs, related to Figures 4 and S6

Table S35. Results of the comparison of the correlation coefficients of personality traits between mother–son dyads and unrelated pairs, related to Figures 4 and S7

Table S36. Results of the comparison of the correlation coefficients of personality traits between mother–daughter dyads and unrelated pairs, related to Figures 4 and S8
