# Parent–offspring brain similarity: Specificities and commonalities among sex combinations–the TRIO study

**DOI:** 10.1016/j.isci.2025.113238

**Published:** 2025-08-26

**Authors:** Izumi Matsudaira, Ryo Yamaguchi, Yasuyuki Taki

## Main text

(iScience *28*, 112936; July 18, 2025)

During copyediting, the production team expanded the definition of the study “transmit radiant individuality to offspring” in the article title rather than maintaining the original desired abbreviation, “TRIO.” This has since been corrected online, and the production team apologizes for any inconvenience.

