# Supplementary material for: Parent–offspring brain similarity: Specificities and commonalities among sex combinations–the TRIO study
Source: iScience. 2025 Jun 19;28(7):112936. doi: 10.1016/j.isci.2025.112936 (PMC12270804; doi:10.1016/j.isci.2025.112936)
Supplement: Document S1. Figures S1–S8 [file mmc1.pdf]

**iScience, Volume *28***

## Supplemental information

# Parent–offspring brain similarity: Specificities and commonalities among sex combinations–the TRIO study

**Izumi Matsudaira, Ryo Yamaguchi, and Yasuyuki Taki**

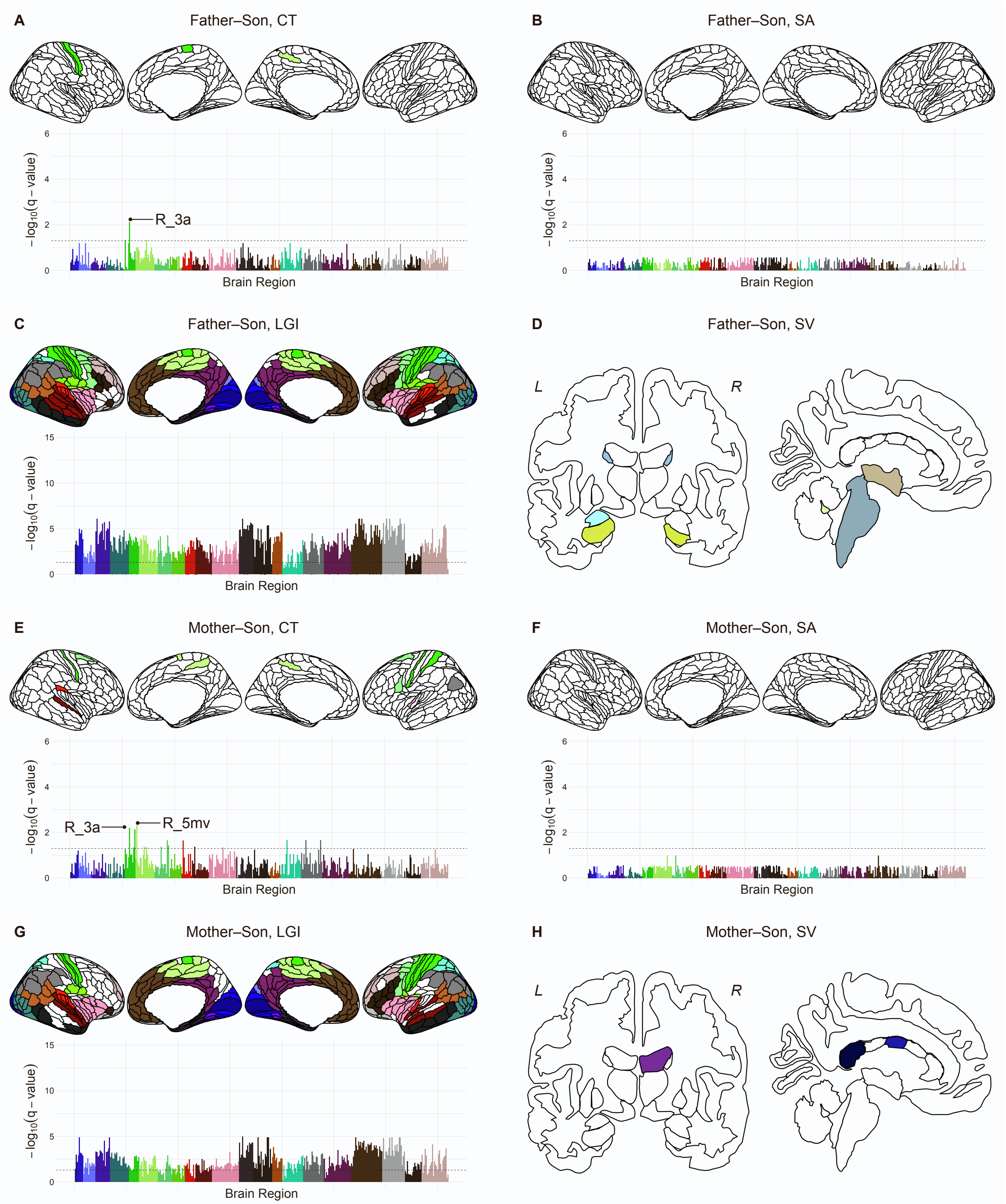


**Figure S1. Neural similarities in father–son dyads and mother–son dyads, related to Figure 3 and Table S9-16.** (A-H) Brain regions showing significantly stronger correlations in CT, SA, LGI, or SV in father–son or mother–son dyads compared to unrelated pairs. The original HCP-MMP1 atlas comprises 180 regions per hemisphere, which were grouped into 22 larger sections. The regions for which significantly stronger correlations were observed in father–offspring or mother–offspring dyads than in unrelated pairs are color-coded. The FDR-corrected *p*-values (*q*-values) for the 360 brain regions are presented in the plot. The vertical axis of the plot represents the log-transformed *q*-values. Each bar represents an individual brain region, and the colors of the bars correspond to those in the brain illustration. The horizontal dashed line represents the significance threshold of a *q*-value < 0.05. Subcortical regions in which significantly stronger correlations were observed between parents and offspring than between unrelated pairs are color-coded according to the ggseg plotting tool. CT, cortical thickness; LGI, local gyrification index; SA, surface area; SV, subcortical volume, L, left hemisphere; R, right hemisphere.

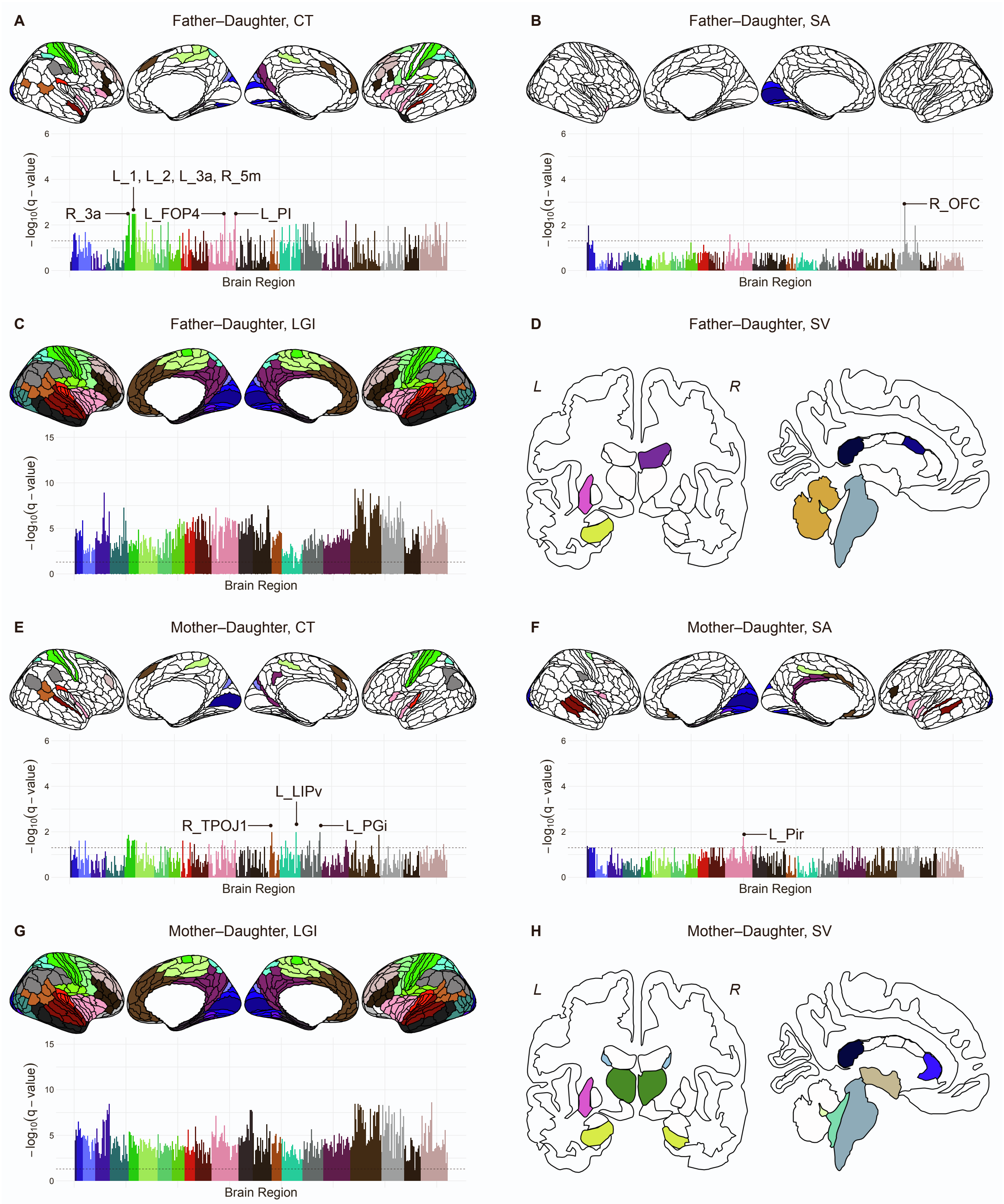


**Figure S2. Neural similarities in father–daughter and mother–daughter dyads, related to Figure 3 and Table S17-24.**

(A-H) Brain regions showing significantly stronger correlations in CT, SA, LGI, or SV in father–daughter or mother–daughter dyads compared to unrelated pairs. The original HCP-MMP1 atlas comprises 180 regions per hemisphere, which were grouped into 22 larger sections. The regions for which significantly stronger correlations were observed in father–offspring or mother–offspring dyads than in unrelated pairs are color-coded. The FDR-corrected *p*-values (*q*-values) for the 360 brain regions are presented in the plot. The vertical axis of the plot represents the log-transformed *q*-values. Each bar represents an individual brain region, and the colors of the bars correspond to those in the brain illustration. The horizontal dashed line represents the significance threshold of a *q*-value < 0.05. Subcortical regions in which significantly stronger correlations were observed between parents and offspring than between unrelated pairs are color-coded according to the ggseg plotting tool. CT, cortical thickness; LGI, local gyrification index; SA, surface area; SV, subcortical volume, L, left hemisphere; R, right hemisphere.

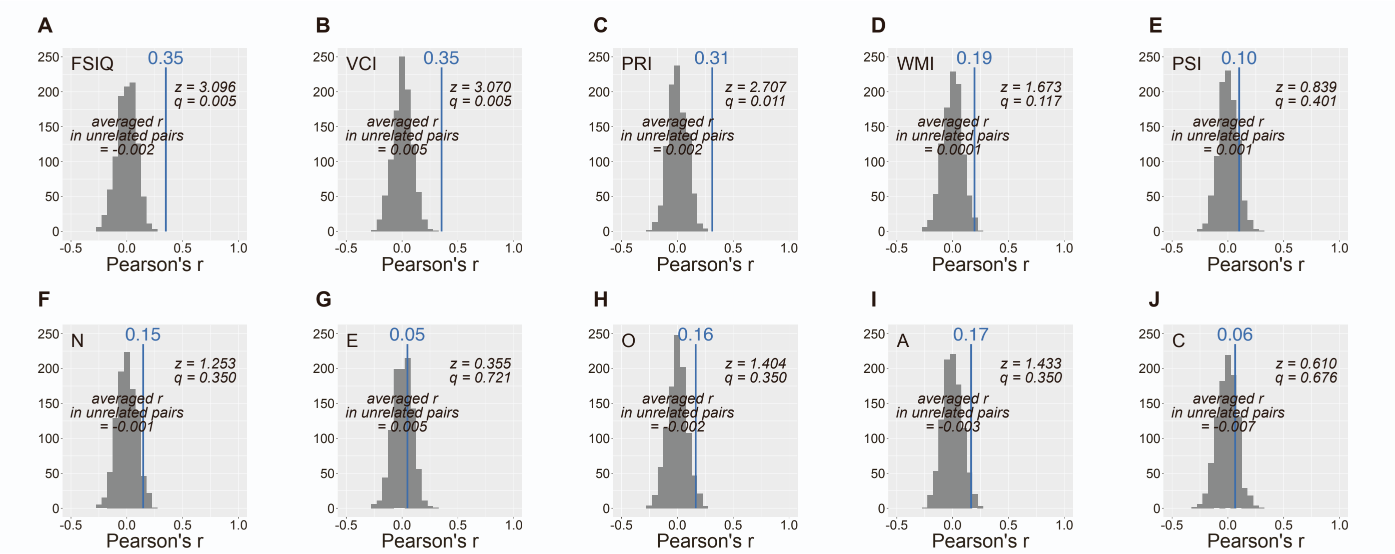


**Figure S3. Behavioral similarities in father–offspring dyads, related to Figure 4 and Table S25, 31.**
(A-J) The differences in correlation coefficients between real father–offspring dyads and unrelated pairs. Gray histograms represent the distribution of correlation coefficients for 1,000 patterns of unrelated pairs. “Averaged r in unrelated pairs” denotes Z-transformed, averaged, and back-transformed correlation coefficients of unrelated pairs. The correlation coefficients for real parent–offspring dyads are presented as solid lines, with the corresponding values labeled above the lines. FSIQ, full-scale intelligence quotient; VCI, verbal comprehension index; PRI, perceptual reasoning index; WMI, working memory index; PSI, processing speed index; N, neuroticism; E, extraversion; O, openness to experience; A, agreeableness; C, conscientiousness.

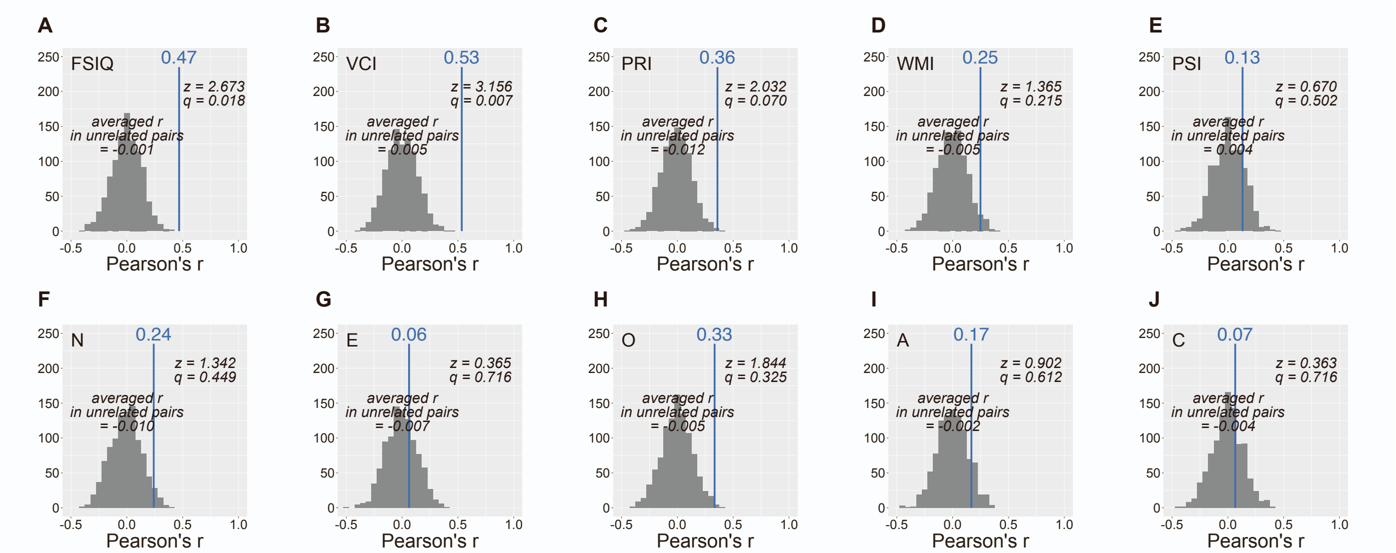


**Figure S4. Behavioral similarities in father–son dyads, related to Figure 4 and Table S26, 32.**
(A-J) The differences in correlation coefficients between real father–son dyads and unrelated pairs. Gray histograms represent the distribution of correlation coefficients for 1,000 patterns of unrelated pairs. “Averaged r in unrelated pairs” denotes Z-transformed, averaged, and back-transformed correlation coefficients of unrelated pairs. The correlation coefficients for real parent–offspring dyads are presented as solid lines, with the corresponding values labeled above the lines. FSIQ, full-scale intelligence quotient; VCI, verbal comprehension index; PRI, perceptual reasoning index; WMI, working memory index; PSI, processing speed index; N, neuroticism; E, extraversion; O, openness to experience; A, agreeableness; C, conscientiousness.

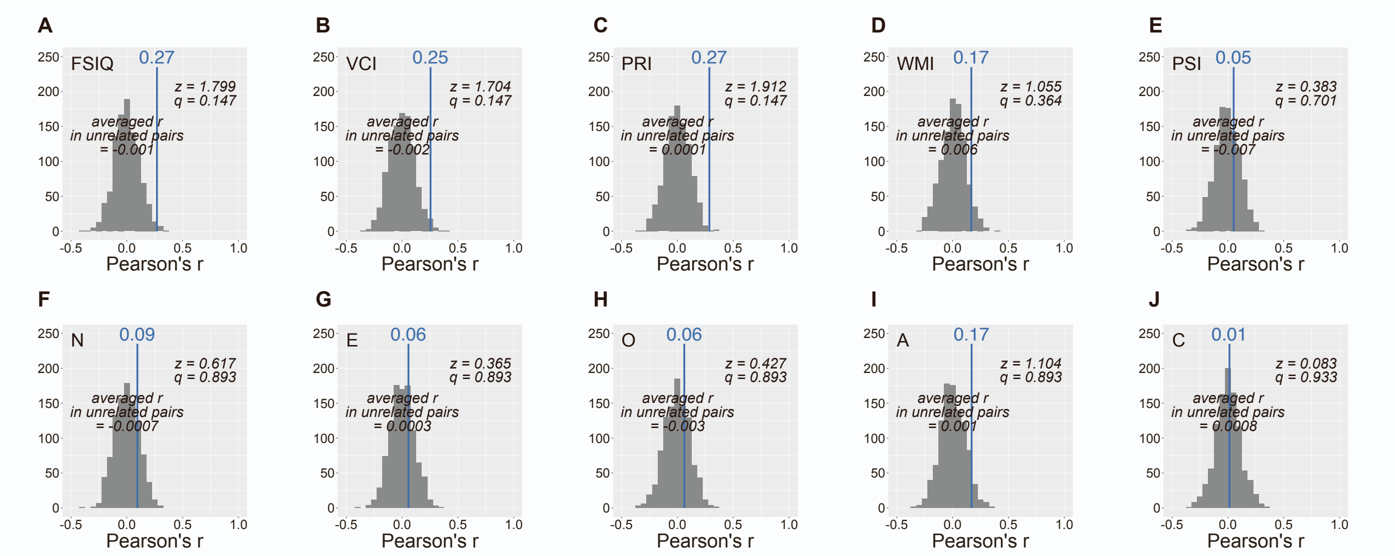


**Figure S5. Behavioral similarities in father–daughter dyads, related to Figure 4 and Table S27, 33.**
(A-J) The differences in correlation coefficients between real father–daughter dyads and unrelated pairs. Gray histograms represent the distribution of correlation coefficients for 1,000 patterns of unrelated pairs. “Averaged r in unrelated pairs” denotes Z-transformed, averaged, and back-transformed correlation coefficients of unrelated pairs. The correlation coefficients for real parent–offspring dyads are presented as solid lines, with the corresponding values labeled above the lines. FSIQ, full-scale intelligence quotient; VCI, verbal comprehension index; PRI, perceptual reasoning index; WMI, working memory index; PSI, processing speed index; N, neuroticism; E, extraversion; O, openness to experience; A, agreeableness; C, conscientiousness.

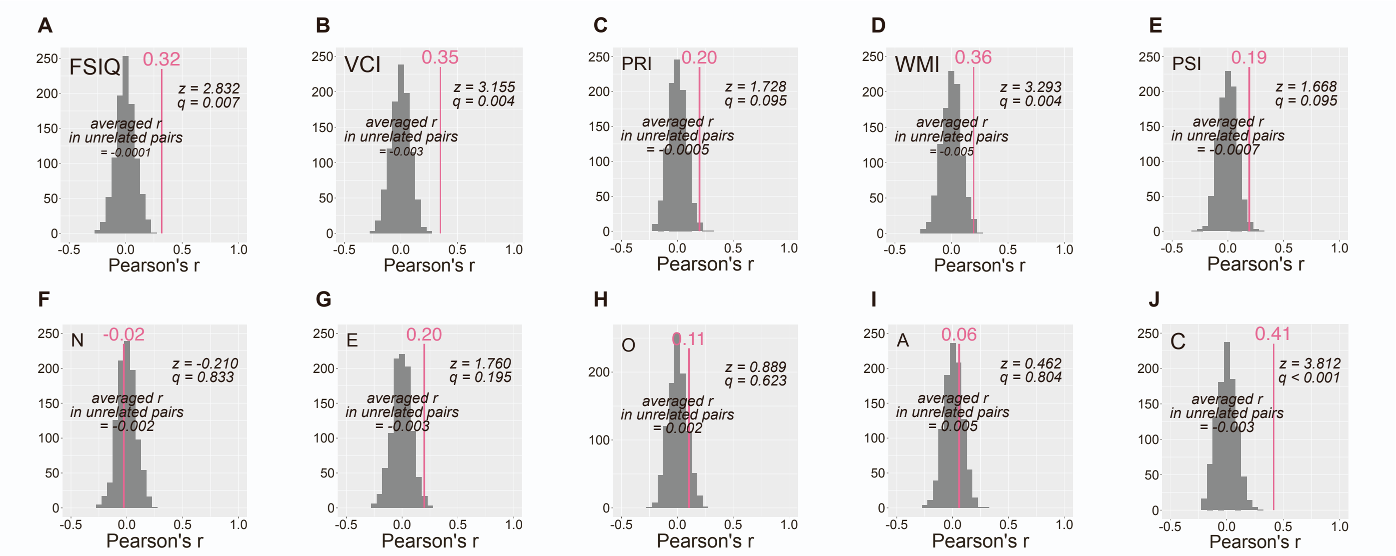


**Figure S6. Behavioral similarities in mother–offspring dyads, related to Figure 4 and Table S28, 34.**
(A-J) The differences in correlation coefficients between real mother–offspring dyads and unrelated pairs. Gray histograms represent the distribution of correlation coefficients for 1,000 patterns of unrelated pairs. “Averaged r in unrelated pairs” denotes Z-transformed, averaged, and back-transformed correlation coefficients of unrelated pairs. The correlation coefficients for real parent–offspring dyads are presented as solid lines, with the corresponding values labeled above the lines. FSIQ, full-scale intelligence quotient; VCI, verbal comprehension index; PRI, perceptual reasoning index; WMI, working memory index; PSI, processing speed index; N, neuroticism; E, extraversion; O, openness to experience; A, agreeableness; C, conscientiousness.

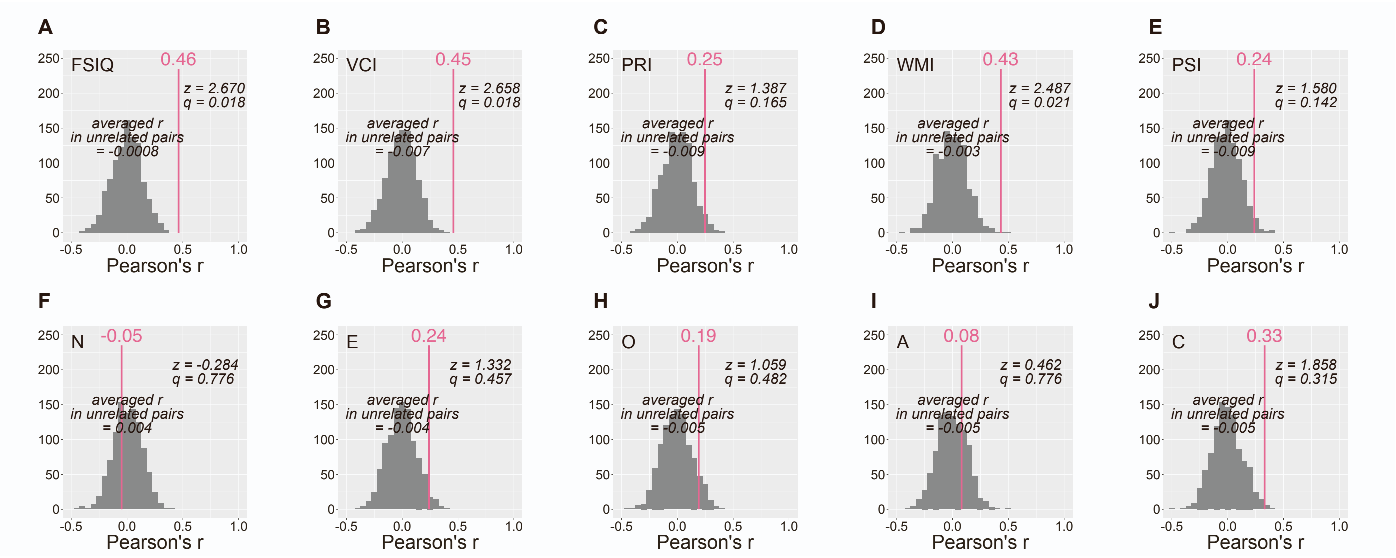


**Figure S7. Behavioral similarities in mother–son dyads, related to Figure 4 and Table S29, 35.**
(A-J) The differences in correlation coefficients between real mother–son dyads and unrelated pairs. Gray histograms represent the distribution of correlation coefficients for 1,000 patterns of unrelated pairs. "Averaged r in unrelated pairs" denotes Z-transformed, averaged, and back-transformed correlation coefficients of unrelated pairs. The correlation coefficients for real parent–offspring dyads are presented as solid lines, with the corresponding values labeled above the lines. FSIQ, full-scale intelligence quotient; VCI, verbal comprehension index; PRI, perceptual reasoning index; WMI, working memory index; PSI, processing speed index; N, neuroticism; E, extraversion; O, openness to experience; A, agreeableness; C, conscientiousness.

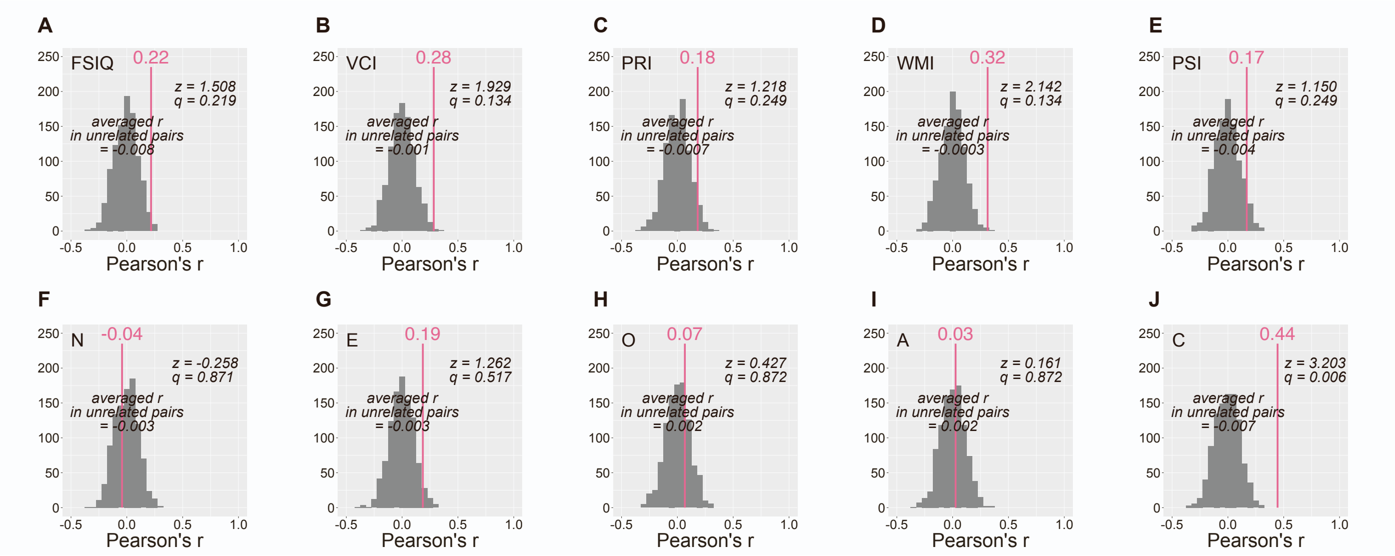


**Figure S8. Behavioral similarities in mother–daughter dyads, related to Figure 4 and Table S30, 36.**
(A-J) The differences in correlation coefficients between real mother–daughter dyads and unrelated pairs. Gray histograms represent the distribution of correlation coefficients for 1,000 patterns of unrelated pairs. “Averaged r in unrelated pairs” denotes Z-transformed, averaged, and back-transformed correlation coefficients of unrelated pairs. The correlation coefficients for real parent–offspring dyads are presented as solid lines, with the corresponding values labeled above the lines. FSIQ, full-scale intelligence quotient; VCI, verbal comprehension index; PRI, perceptual reasoning index; WMI, working memory index; PSI, processing speed index; N, neuroticism; E, extraversion; O, openness to experience; A, agreeableness; C, conscientiousness.
